# New records of spider wasps (Hymenoptera, Pompilidae) from Colombia

**DOI:** 10.3897/zookeys.443.8348

**Published:** 2014-09-29

**Authors:** Valentina Castro-Huertas, James P. Pitts, Juanita Rodriguez, Cecilia Waichert, Fernando Fernández

**Affiliations:** 1 Instituto de Ciencias Naturales, Universidad Nacional de Colombia, Bogotá, Colombia; 2 Department of Biology, Utah State University, Logan, UT, USA; 3 Department of Biological Sciences, Auburn University, Auburn, AL, USA

**Keywords:** Pompilidae, new record, Neotropical, South America

## Abstract

New records of genera and species of spider wasps (Hymenoptera: Pompilidae) from Colombia are provided. *Agenioideus*, *Cryptocheilus*, *Evagetes*, *Mystacagenia*, and *Xerochares* are newly recorded genera from Colombia. Nineteen species are first recorded from Colombia: *Aimatocare vitrea* (Fox); *Ageniella azteca* (Cameron); *Ageniella curtipinus* (Cameron); *Ageniella fallax* (Arlé); *Ageniella hirsuta* Banks; *Ageniella pilifrons* (Cameron); *Ageniella pretiosa* Banks; *Ageniella sanguinolenta* (Smith); *Ageniella zeteki* (Banks); *Agenioideus birkmanni* (Banks); *Aporus (Aporus) cuzco* Evans; *Aporus (Cosmiaporus) diverticulus* (Fox); *Aporus (Notoplaniceps) canescens* Smith; *Euplaniceps exilis* (Banks); *Euplaniceps herbertii* (Fox); *Irenangelus clarus* Evans; *Mystacagenia bellula* Evans; *Phanochilus nobilitatus* (Smith) and *Xerochares expulsus* Schulz. The following species and genera have their occurence ranges expanded for South America: *Ageniella azteca* (Cameron); *Ageniella zeteki* (Banks); *Agenioideus birkmanni* (Banks); and *Xerochares expulsus* Schulz; *Cryptocheilus* Panzer; and *Xerochares* Evans.

## Introduction

Spider wasps (Hymenoptera: Pompilidae) are a widespread group of about 5,000 described species (Pitts et al. 2006) in approximately 120 genera (Wasbauer 1995). In the Neotropical region there are four subfamilies, 60 genera, and about 1,000 species (Fernández 2000, Hanson and Wasbauer 2006). The taxonomy and systematics of pompilids are poorly understood, making it one of the most taxonomically challenging families of Hymenoptera. Future work should be focused on making extensive collections and appropriate identifications to get a better understanding of their taxonomy.

The fauna of spider wasps from Colombia and South America is poorly known and not fully explored. Colombia has about 29 genera and approximately 143 species of spider wasps described (Fernández 2000). As part of a project on the systematics of Pompilidae from Colombia and northern South America, we offer new records of genera and species for these regions.

## Materials and methods

*Examined specimens.* When citing the examined material, a backslash indicates a separate label. The specimens are deposited in the Entomological collection of the Instituto Alexander von Humboldt, Villa de Leiva, Colombia (IAvH-E), and in the Instituto de Ciencias Naturales of the Universidad Nacional de Colombia, Bogotá, Colombia (ICN). Specimens from the Utah State University Entomology Collection, Logan, UT (EMUS) were examined for *Agenioideus birkmanni* and *Phanochilus nobilitatus*.

## Results

The following species and genera are reported for the first time for Colombia.

### Subfamily Ceropalinae

***Irenangelus clarus* Evans, 1969**

**Specimen data.** BOLIVAR. SFF Los Colorados, Alto el Mirador, 9°54'N, 75°7'W], 400m, malaise, 4–30 ene 2002, E. Deulufeut, M2935 / IAvH-E 107755 (1m, IAvH-E).

**Comments.** This species has been recorded for Brazil and Argentina (Fernández 2000).

### Subfamily Pepsinae

***Aimatocare vitrea* (Fox, 1897)**

**Specimen data.** RISARALDA. SFF Otún Quimbaya, Robledal, [4°44'N, 75°35'W], 1980m, malaise, 4–18 ene 2003, G. López, M3704, (1m, IAvH-E); 18 feb–4 mar 2003, G. López, M3699, (1m, IAvH-E). VAUPÉS. Estación Mosiro-Itajura (Caparú), Centro Ambiental, [1°4'S, 69°31'W], 60m, malaise, 9–25 feb 2003, J. Pinzón, M3639, (1f, IAvH-E).

**Comments.** This species has been recorded for Brazil and British Guiana (Fernández 2000).

***Ageniella azteca* (Cameron, 1891)**

**Specimen data.** TOLIMA. Armero-Guayabal, Hda. Cardonal, [5°5'385"N, 74°46'395"W], 350m, 17 nov 1995, F. Fernández / IAvH-E 107940, (1f, IAvH-E).

**Comments.** New record for South America. This species was first described for Mexico and later recorded for Nicaragua (Wahis 1995).

***Ageniella curtipinus* (Cameron, 1912)**

**Specimen data.** BOLÍVAR. Zambrano, Hda. Monterrey, [9°45'N, 74°49'W], 10m, malaise, 29 may 1993, F. Fernandez / IAvH-E 107937, (1f, IAvH-E).

**Comments.** This species has been recorded for Bristih Guiana, Peru, and Trinidad (Wahis 1995).

***Ageniella fallax* (Arlé, 1947)**

**Specimen data.** BOLÍVAR. SFF Los Colorados, Diana, [9°54'N, 75°7'W], 150m, malaise, 15–30 sep 2000, E. Deulufeut / IAvH-E 107760, (1m, IAvH-E).

**Comments.** This species has been recorded for Argentina, Brazil, Peru (Fernández 2000), and Panama (Cambra 2005).

***Ageniella hirsuta* Banks, 1946**

**Specimen data.** PUTUMAYO. PNN La Paya, Loma Alta, [0°6'S, 74°58'W], 350m, malaise, 18 jun-1 jul 2003, R. Cobete, (1f, IAvH-E).

**Comments.** This species has been recorded for Ecuador (Fernández 2000).

***Ageniella pilifrons* (Cameron, 1912)**

**Specimen data.** CAUCA. PNN Gorgona. [2°58'N, 78°11'W], 60m, malaise, 3–18 ene 2001, H. Torres, M1235 / IAvH-E 107720, (1f, IAvH-E). META. PNN Sierra de la Macarena, Cerro El Tablazo, [3°20'N, 73°56'W], 486m, manual, 26 dic 2001, D. Campos, M2614 / IAvH-E 10760 (1f, IAvH-E); Caño La Curia, 580m, 23 dic 1993, con Heteropodidae, F. Fernández / IAvH-E 107942, (1 f, IAvH-E); Cabaña Cerrillo, [3°21'N, 73°56'W], 460m, malaise, 4–17 ene 2003, A.Herrera & W. Villalba, (1f, IAvH-E).

**Comments.** This species has been recorded for British Guiana (Fernández 2000).

***Ageniella pretiosa* Banks, 1946**

**Specimen data.** AMAZONAS. PNN Amacayacu. San Martín, [3°23'S, 70°6'W], 150m, malaise, 2–7 jul 2000, B. Amado / IAvH-E 107614, (1f, IAvH-E); Mocagua, [3°23'S, 70°6'W], 150m, malaise, 12–19 jun 2000, A. Parente / IAvH-E 107619, (1f, IAvH-E); VICHADA, PNN Tuparro, Cerro Tomás, [5°21'N; 67°51'W], 140m, malaise, 17–26 dic 2000, W. Villalba / IAvH-E 107628 / IAvH-E 107629, (2f, IAvH-E).

**Comments.** This species has been recorded for Peru (Fernández 2000).

***Ageniella sanguinolenta* (Smith, 1864).**

**Specimen data.** AMAZONAS. PNN Amacayacu, San Martín, [3°23'S, 70°6'W], 150m, malaise, 5–19 nov 2001, D. Chota / IAvH-E 107618, (1 f, IAvH-E); BOLÍVAR. SFF Los Colorados, Diana, [9°54'N, 75°7'W], 150m, Malaise, 16–30 nov 2000, E. Deulufeut / IAvH-E 107756, (1f, IAvH-E); CAUCA. PNN Gorgona, El Helechal, 2°58'N, 78°11'W, 30m, malaise, 9–27 ago 2001, H. Torres / IAvH-E 107710 (1f, IAvH-E).

**Comments.** This species has been recorded for Brazil (Smith 1864).

***Ageniella zeteki* (Banks, 1925)**

**Specimen data.** CAUCA. PNN Gorgona, Antigua Laguna, [2°58'N, 78°11'W), 70m, malaise, 18 jul-16 ago 2000, H. Torres / IAvH-E 107697 (1f, IAvH-E); El Helechal, [2°58'N, 78°11'W], 30m, malaise, 12–28 sep 2001, H. Torres / IAvH-E 107696, (1f, IAvH-E). CHOCÓ. PNN Utría, Cocalito Dosel, [6°1'N, 77°, 20'W], 20m, malaise, 30 jul–16 ago 2000, J. Pérez / IAvH-E 107762 (1f, IAvH-E). VICHADA. PNN Tuparro. Centro administrativo, [5°21'N, 67°51'W], 100m, malaise, 22 may-3 jun 2001, I. Gil / IAvH-E 107632, (1f, IAvH-E).

**Comments.** New record for South America. This species was previously recorded for Panama (Banks 1925, Cambra et al. 2004).

***Cryptocheilus* sp.**

**Specimen data.** BOYACÁ. Sotaquirá, Hueco, 1 abr 1969, J. R. Alba, (1m, ICN).

**Comments.** New record for South America. *Cryptocheilus* Panzer, 1806 is a cosmopolitan genus with six species described from the Nearctic Region and two from the Neotropical region: *Cryptocheilus neotropicalis* is found in Panama and *Cryptocheilus santosi* is found in Costa Rica and Panama (Cambra and Wahis 2005). This species does not fit the diagnosis of any of the Central American species. The specimen studied herein is similar to *Cryptocheilus pallidipennis* (Banks, 1912), but this species has Nearctic distribution (Townes^ 1957^). A revision of the New World species of the genus is needed to determine if the studied specimen is an undescribed species or not.

***Mystacagenia bellula* Evans, 1973**

**Specimen data.** CUNDINAMARCA, PNN Sumapaz, Cabañas Las Mirlas, [4°30'N, 73°45'W], malaise, 3–30 abr 2002, H. Vargas / IAvH-E 107683, (1f, IAvH-E); [4°48'N, 73°52'W], 710m, malaise, 19 mar–3 abr 2002, H. Vargas, (1f, IAvH-E). META. PNN Sierra de la Macarena, Caño Curia, Sendero Cachicamos, [3°21'N, 73°56'W], 460m, malaise, 9–24 feb 2003, W. Villalba, (1f, IAvH-E); Villavicencio, Vda. Vanguardia, Pozo Azul, malaise, 16–17 abr 2005, Sistematica Animal, (1f, ICN).

**Comments.** The genus, known only from females, is rarely collected and comprises four known species: *Mystacagenia variegata* Evans (Brazil), *Mystacagenia bellula* Evans (Peru), *Mystacagenia albiceps* Evans (Peru) and *Mystacagenia elengatula* Evans (Panama) (Evans 1973, 1980).

***Phanochilus nobilitatus* (Smith, 1864)**

**Specimen data.** Amazonas. Amacayacu, Mata-Mata, 03˚48'36"S, 70˚20'57"W, malaise, ii.1989, F. Fernández (1f, IAvH 107951). META. PNN Sierra de La Macarena, Cabaña Cerrillo, 3°21'N 73°56'W, 480 m, malaise trap, 10.xi–21.xii.2002, A. Herrera & W. Villalba leg. M.2982 (1f, EMUS).

**Comments.** This species has been recorded for British Guiana and Peru (Fernández 2000).

### Subfamily Pompilinae

***Agenioideus birkmanni* (Banks, 1910)**

**Specimen data.** MAGDALENA. PNN Tayrona, Palangana, [11°20'N, 74°2'W], 30m, malaise, 31 ene–15 feb 2002, R. Henríquez, M3032, (2m, IAvH-E).

**Comments.** New record for South America. This species has been previously recorded from northern Oregon east to New Jersey and south to Oaxaca, Mexico (Wasbauer and Kimsey 1985). Specimens from Costa Rica are found in EMUS. This record represents a considerable range extension of what was previously known as a Nearctic species.

***Xerochares expulsus* Schulz, 1906**

**Specimen data.** MAGDALENA. PNN Tayrona, Neguanje, [11°20'N, 74°2'W]; 10m, malaise, 17–27 sep 2001, R. Henríquez, (13m, 1f IAvH-E); 28 jul–18 ago 2001, R. Henriquez, (10m, 1f, IAvH-E); Palangana, [11°20'N, 74°2'W], 30m, malaise, 4–23 may 2001, R. Henríquez, (5m, IAvH-E).

**Comments.** New record for South America. The species was previously known from southern New Mexico to western Mexico to Guatemala, El Salvador, and Nicaragua (Evans 1966; Wasbauer and Kimsey 1985).

***Evagetes* sp.**

**Specimen data.** BOYACÁ. SFF Iguaque, Cabana Chaina, [5°25'N, 73°27'W], 2,600m, malaise, 9–26 sep 2002, A. Roberto, (22m, IAvH-E).

**Comments.** *Evagetes* Lepeletier, 1845 is a cleptoparasitic genus rarely collected and poorly known in the Neotropics, with four described species from Southern South America (Evans 1966, Fernández 2000). There is no recent taxonomic revision of the genus, therefore, the identification at the species-level is challenging.

**Aporus (Aporus) cuzco Evans, 1973**

**Specimen data.** BOYACÁ. SFF Iguaque, Cabaña Chaina, [5°25'N, 73°27'W], 2,600m, malaise, 16 sep–6 oct 2001, P. Reina, M2199 / IAvH-E 107986, (1m, IAvH-E); 9–26 sep 2002, A. Roberto, M3332, (1m, IAvH-E); 6–25 oct 2001, P. Reina, M2477, (1m, IAvH-E); Villa de Leyva, El Roble, 2,200m, red, 9 jun 2001, D. Campos, et al. / IAvH-E 108278, (1f, IAvH-E). CAQUETÁ. PNN Picachos, [2°44'N, 74°53'W], 1,560m, malaise, 1–7 nov 1997, E. González / IAvH-E 108219, (1f, IAvH-E). CUNDINAMARCA. PNN Sumapaz, Jardín Botánico, [4°30'N, 73°45'W], malaise, 4–24 ene 2002, H. Vargas, M3109 / IAvH-E 107989, (1f, IAvH-E); [3°48'N, 73°56'W], 730m, malaise, 4–24 ene 2002, H. Vargas, M3109, (1m, IAvH-E). HUILA. PNN Cueva de los Guacharos, Alto el Mirador, [1°38'N, 76°6'W], 1,980m, malaise, 6–21 abr 2002, J. Fonseca, M3127, M3258, (2f, IAvH-E); 4–18 feb 2002, F. Quevedo / IAvH-E 108187, (1f, IAvH-E). MAGDALENA. Santa Marta, Ciudad Perdida, [11°14'50"N, 74°12'6"W], 1,200m, 25–26 nov 1995, A. Amarillo, CES260 / IAvH-E 108244, (1f, IAvH-E); PNN Tayrona, Cerro San Lucas, La Antena, [11°20'N, 74°2'W], 700m, malaise, 19–24 jul 2002, M. Sharkey, D. Arias & E. Torres, (1m, IAvH-E). NARIÑO. R.N. La Planada, Parcela Olga, [1°15'N, 78°15'W], 1,850m, malaise, 29 feb-14 mar 2004, G. Olivia, M4710, (1f, IAvH-E); Centro Administrativo, [1°15'N, 78°15'W], 1,700m, red, 9–12 ago 2004, D. Arias, M4901, (1m, IAvH-E). NORTE DE SANTANDER. ANU Los Estoraques, Platanillo, [8°13'N, 73°14'W], 1,516m, malaise, 1–15 sep 2003, J. Vargas, M4087, (1m, IAvH-E). QUINDÍO. Circasia, Vda. Buenavista, Fca. Calamar, [4°36'53"N, 75°41'56"W], 1,460m, malaise, 10 oct 1999, E. González / IAvH-E 108262, (1m, IAvH-E). RISARALDA. SFF Otún Quimbaya, Cuchilla camino, [4°43'N, 75°35'W], 2,050m, malaise, 26 sep-11 oct 2003, G. López, M4212, (1f, IAvH-E); Robledal, [4°44'N, 75°35'W], 1,980m, malaise, 4–18 ene 2003, G. López, M3704, (1f, IAvH-E); 20 dic 2002–3 ene 2003, R. Walker, M2974, (1f, IAvH-E). VALLE DEL CAUCA. PNN Farallones de Cali, Cgto. Los Andes, Vda. Quebradahonda, [3°34'N, 76°40'W], 1,730m, malaise, 26 ago 1998, N. Beltrán & H. Peña / IAvH-E 108253, (1f, IAvH-E); La Meseta, [3°34'N, 76°40'W], 2,080m, malaise, 24 dic 2003–27 ene 2004, S. Sarria & M. Losso, M4553, (1f, IAvH-E).

**Comments.** This species has been recorded for Peru (Fernández 2000).

**Aporus (Cosmiaporus) diverticulus (Fox, 1897)**

**Specimen data.** HUILA. PNN Cueva de los Guacharos, Alto el Mirador, [1°38'N, 76°6'W], 1,980m, malaise, 21 abr–5 may 2002, J. Fonseca, M3128, (1m, IAvH-E); PUTUMAYO. PNN La Paya, Cabaña Chagra, [0°7'S, 74°56'W], 320m, malaise, 16–30 dic 2001, E. Lozano / IAvH-E 108201, (1f, IAvH-E).

**Comments.** This species has been recorded for Brazil (Fernández 2000).

**Aporus (Notoplaniceps) canescens Smith, 1873**

**Specimen data.** AMAZONAS. PNN Amacayacu, Matamata, [3°41'S, 70°15'W], 150m, malaise, 24 mar–3 abr 2000, A. Parente, M38, (1m, IAvH-E); San Martín, [3°23'S, 70°6'W], 150m, malaise, 22–30 ene 2001, B. Amado / IAvH-E 108233, (1f, IAvH-E). CAQUETÁ. Santa Rosita, [1°20'N, 76°6'W], 600m, malaise, 22 jul-4 ago 2000, F. Ruales / IAvH-E 108212, (1f, IAvH-E). MAGDALENA, PNN Tayrona, Cerro San Lucas, La Antena, 11°20'N, 74°2'W, 700m, malaise, 19–24 jul 2002, M. Sharkey, D. Arias & E. Torres, M3258, (1m, IAvH-E). META. PNN Sumapaz, Qda. La Cristalina, [3°48'N, 73°56'W], 614m, malaise, 20 ago-5 sep 2003, H. Vargas & A. Torrijos, M4342, (1f, IAvH-E); PNN Sierra de la Macarena, cabaña Cerrillo, [3°21'N, 73°56'W], 460m, malaise, 10 nov-21 dic 2002, A. Herrera &W Villalba, M2982, (1f, IAvH-E); PNN Tinigua, Vda. Bajo Raudal, malaise, 5–19 ene 2003, C. Sánchez, M3477, (1m, IAvH-E). PUTUMAYO. PNN La Paya, cabaña Viviano, [0°7'S, 74°56'W], 320m, malaise, 18–21 abr 2002, R. Cobete, M3252, (1f, IAvH-E). RISARALDA. SFF Otún Quimbaya, Robledal, [4°44'N, 75°35'W], 1.980m, malaise, 4–18 ene 2003, G. López, M3704, (1m, IAvH-E); VAUPÉS. Estación Mosiro-Itajura (Caparu), Centro Ambiental, [1°4'S, 69°31'W], 60m, malaise, 9–25 feb 2003, J. Pinzón, M3639, (1m, IAvH-E); fit, 20 ene-1 feb 2003, M. Sharkey & D. Arias, M3388, (IAvH-E); VICHADA. PNN El Tuparro, Cerro Tomás, [5°21'N, 67°51'W], 140m, malaise, 27 dic 2000–5 ene 2001, M955, W. Villalba, (1f, IAvH-E).

**Comments.** This species has been recorded for Trinidad and Tobago, Argentina, Brazil, and Peru (Fernández 2000).

***Euplaniceps exilis* (Banks, 1944)**

**Specimen data.** CAQUETÁ. PNN Serranía de Chiribiquete, Caño-Amú, Bos.inundable y tierra firme, [0°14'N, 72°27'W], 300m, malaise, 21–25 nov 2000, E. González & M. Ospina, M961, (1m, IAvH-E); Puerto Abeja, [0°4'N, 72°26'W], 310m, 29 oct-12 nov 2000, J. Forero, M955, (1m, IAvH-E). MAGDALENA. PNN Tayrona, Zaino, [11°20'N, 74°2'W], 50m, malaise, 14–30 jul 2000, R. Henriquez, M564, (1m, IAvH-E). META. PNN Sierra de la Macarena, Caño Curia, Sendero Cachicamos, [3°21'N, 73°38'W], 493m, malaise, 13–30 sep 2004, W. Villalba, (1m, IAvH-E). VAUPÉS, RN Mosiro-Itajura (Caparú), Centro Ambiental, [1°4'S, 69°31'W], 60m, malaise, 20 ene–1 feb 2003, M. Sharkey & D. Arias, M3386, (1m, IAvH-E); Antigua Cabaña, [1°4'N, 69°3'W], 60m, malaise, 17–29 abr 2003, J. Pinzón, M3615, (IAvH-E). VICHADA. PNN El Tuparro, Cerro Tomás, [5°21'N, 67°51'W], 140m, malaise, 17–26 dic 2000, W. Villalba, M1386, (2m, IAvH-E); Centro Administrativo, [5°21'N, 67°51'W], 100m, 15–19 jul 2000, M510, W. Villalba, (1m, IAvH-E).

**Comments.** This species has been recorded for Guiana and Suriname (Banks 1944, Cambra et al. 2013)

***Euplaniceps herbertii* (Fox, 1897)**

**Specimen data.** MAGDALENA. PNN Tayrona, Cerro San Lucas, [11°19'N, 73°53'W], 400m, malaise, 6–11 nov 2003, C. Sarmiento, (1f, IAvH-E). META. PNN Sierra de la Macarena, Caño Curia, Sendero Cachicamos, [3°21'N, 73°56'W], 460m, malaise, 21 dic 2002–4 ene 2003, M. Duarte, (1f, IAvH-E); 10 nov-21 dic 2002, M. Duarte, (1f, IAvH-E).

**Comments.** This species has been recorded for Guiana and Brazil (Fernández 2000, Cambra et al. 2013).
